# Development of a Reactive Stroma Associated with Prostatic Intraepithelial Neoplasia in EAF2 Deficient Mice

**DOI:** 10.1371/journal.pone.0079542

**Published:** 2013-11-18

**Authors:** Laura E. Pascal, Junkui Ai, Khalid Z. Masoodi, Yujuan Wang, Dan Wang, Kurtis Eisermann, Lora H. Rigatti, Katherine J. O’Malley, Hei M. Ma, Xinhui Wang, Javid A. Dar, Anil V. Parwani, Brian W. Simons, Michael M. Ittman, Luyuan Li, Benjamin J. Davies, Zhou Wang

**Affiliations:** 1 Department of Urology, University of Pittsburgh School of Medicine, Pittsburgh, Pennsylvania, United States of America; 2 Division of Laboratory Animal Resources, University of Pittsburgh School of Medicine, Pittsburgh, Pennsylvania, United States of America; 3 Surgical Oncology, Massachusetts General Hospital, Harvard Medical School, Boston, Massachusetts, United States of America; 4 Department of Pathology, University of Pittsburgh School of Medicine, Pittsburgh, Pennsylvania, United States of America; 5 Department of Molecular and Comparative Pathobiology, Johns Hopkins University School of Medicine, Baltimore, Maryland, United States of America; 6 Department of Pathology and Immunology, Baylor College of Medicine, Houston, Texas, United States of America; 7 College of Pharmacy, Nankai University, Tianjin, China; 8 University of Pittsburgh Cancer Institute, University of Pittsburgh School of Medicine, Pittsburgh, Pennsylvania, United States of America; 9 Department of Pharmacology and Chemical Biology, and University of Pittsburgh School of Medicine, Pittsburgh, Pennsylvania, United States of America; Rutgers University -New Jersey Medical School, United States of America

## Abstract

ELL-associated factor 2 (EAF2) is an androgen-responsive tumor suppressor frequently deleted in advanced prostate cancer that functions as a transcription elongation factor of RNA Pol II through interaction with the ELL family proteins. EAF2 knockout mice on a 129P2/OLA-C57BL/6J background developed late-onset lung adenocarcinoma, hepatocellular carcinoma, B-cell lymphoma and high-grade prostatic intraepithelial neoplasia. In order to further characterize the role of EAF2 in the development of prostatic defects, the effects of EAF2 loss were compared in different murine strains. In the current study, aged EAF2^−/−^ mice on both the C57BL/6J and FVB/NJ backgrounds exhibited mPIN lesions as previously reported on a 129P2/OLA-C57BL/6J background. In contrast to the 129P2/OLA-C57BL/6J mixed genetic background, the mPIN lesions in C57BL/6J and FVB/NJ EAF2^−/−^ mice were associated with stromal defects characteristic of a reactive stroma and a statistically significant increase in prostate microvessel density. Stromal inflammation and increased microvessel density was evident in EAF2-deficient mice on a pure C57BL/6J background at an early age and preceded the development of the histologic epithelial hyperplasia and neoplasia found in the prostates of older EAF2^−/−^ animals. Mice deficient in EAF2 had an increased recovery rate and a decreased overall response to the effects of androgen deprivation. EAF2 expression in human cancer was significantly down-regulated and microvessel density was significantly increased compared to matched normal prostate tissue; furthermore EAF2 expression was negatively correlated with microvessel density. These results suggest that the EAF2 knockout mouse on the C57BL/6J and FVB/NJ genetic backgrounds provides a model of PIN lesions associated with an altered prostate microvasculature and reactive stromal compartment corresponding to that reported in human prostate tumors.

## Introduction

The prostate gland is tightly regulated by androgens and consists of acini lined by secretory luminal cells and an underlying layer of basal cells embedded within a fibromuscular stroma. In the normal prostate, stromal cells direct epithelial differentiation and development through growth factors and androgen stimulation [1]. During prostate carcinogenesis, the stromal cells immediately adjacent to prostate tumor cells are characterized by a modified extracellular matrix, increased microvessel density and a myofibroblastic phenotype [2]. The evolution of this reactive stroma and its role in prostate disease development is not yet fully understood, however, evidence of altered stroma has been identified in precancerous prostatic intraepithelial neoplasia (PIN) lesions as well as in prostate tumors [3], [4]. Greater understanding of the role of genes in regulating the stromal and vascular microenvironment could provide insight into the mechanisms of early prostate disease development as well as progression and metastasis.

ELL-associated factor 2 (EAF2) is a potential tumor suppressor gene that was found to be up-regulated in response to androgens in the rat ventral prostate [5] and in the human prostate cancer cell line LNCaP [6]. EAF2 interacts with the RNA polymerase II (pol II) elongation factor eleven-nineteen lysine-rich in leukemia (ELL) in the regulation of transcription elongation [7]. EAF2 protein down-regulation, allelic loss, promoter hypermethylation and possibly homozygous deletion was identified in ∼80% of advanced prostate cancer specimens examined (Gleason ≥7) [6]. Overexpression of EAF2 in prostate cancer cell lines induced apoptosis and inhibited the growth of xenograft tumors [6]. EAF2 knockout mice developed lung adenocarcinoma, hepatocellular carcinoma, B-cell lymphoma and high-grade murine prostatic intraepithelial neoplasia (mPIN) [8]. In addition, EAF2 loss in a murine model was found to increase microvessel density in aged animals and to enhance the angiogenic effects of Von Hippel-Lindau (VHL) heterozygosity in the liver and prostate [9]. Prostates of aged EAF2^−/−^ VHL^+/−^ mice displayed increased mPIN, stromal inflammation, fibrosis and smooth muscle proliferation. PIN and hepatic vascular lesions in EAF2^−/−^ VHL^+/−^ animals were characterized by decreased expression of pVHL, suggesting that EAF2 enhanced the angiogenic effects of VHL heterozygosity [9].

EAF2 has also recently been shown to regulate the potent anti-angiogenic factor thrombospondin 1 (TSP-1) through interaction with the tumor suppressor p53, suggesting a role for EAF2 in TSP-1 regulated angiogenesis [10]. TSP-1 inhibits the proliferation and migration of endothelial cells and increases apoptosis [11]. Furthermore, decreased expression of TSP-1 has been reported in prostate cancer [12]. In androgen-dependent prostate tumors, TSP-1 expression is inversely correlated with microvessel density (MVD) [13]–[15]. In the prostates of young EAF2 knockout animals, TSP-1 expression was significantly reduced compared to wild-type controls [10]. Additionally, overexpression of EAF2 and p53 in LNCaP and H1299 cells increased TSP-1 promoter activity, suggesting that EAF2 and p53 could functionally interact in the repression of angiogenesis through up-regulation of TSP-1 [10]. Interaction of EAF2 with its binding partners p53 and VHL in the regulation of pro-angiogenic HIF1α and anti-angiogenic TSP-1 may be critical for maintaining normal prostate homeostasis. Dysregulation of these interacting pathways could promote increased vascularization, cellular proliferation and prostate tumorigenesis.

In the current study, the relationship between EAF2 loss and the development of PIN lesions and increased microvasculature was further explored. EAF2 knockout mice were generated on a pure C57BL/6J or a pure FVB/NJ background and examined at various ages for histological defects in the prostate. EAF2-deficiency on either background was associated with an increase in epithelial cell proliferation, the development of mPIN lesions as well as an associated increased incidence in reactive stroma and increased vascularity. Prostate stromal cells isolated from EAF2^−/−^ animals at age 15 mos were characterized by decreased growth rate compared to wild-type controls. Furthermore, EAF2 expression was negatively correlated with microvessel density in human prostate tissue specimens. These findings suggest that the development of PIN lesions in the murine EAF2^−/−^ mouse is associated with the development of a reactive stroma that is similar to that reported in human prostate cancer.

## Materials and Methods

### Generation of EAF2 Deletion Mice

Preparation of mice with specific deletion of the EAF2 gene has been described previously [8], [10]. Heterozygous EAF2^+/−^ mice were initially generated using HM1 embryonic stem cells [8], and were subsequently backcrossed to the C57BL/6J and FVB/NJ strains (The Jackson Laboratory, Bar Harbor, ME, USA) for >12 generations to generate EAF2^−/−^ mice with a pure C57BL/6J and a pure FVB/NJ background. Experimental cohorts were wild type and homozygous C57BL/6J and FVB/NJ male littermates, all mice were maintained identically. All animal studies were reviewed and approved by the Institutional Animal Care and Use Committee (IACUC) of the University of Pittsburgh and were conducted in strict accordance with the standards for humane animal care and use as set by the Animal Welfare Act and the National Institutes of Health guidelines for the use of laboratory animals. Genotyping was determined by PCR analysis of mouse tail genomic DNA and confirmed on muscle DNA when animals were euthanized as described [8]. Mice on a C57BL/6J background were euthanized at 7 weeks (n = 18 males), 19 weeks (n = 18 males), 12 mos (n = 18 males) and 20–24 mos (n = 19) of age. Mice on an FVB/NJ background were euthanized for histological analysis at 15–20 mos (n = 8). In order to determine the effect of EAF2 loss on response to androgen deprivation, an additional cohort of wild type and EAF2^−/−^ mice on a pure C57BL/6J background were subjected to bilateral orchiectomy at 19 weeks of age and euthanized 3 days (n = 6) and 14 days post-castration (n = 9). Tissue necropsy was performed and organs were cleaned of excess fat and membrane with phosphate-buffered saline; mass was determined after blotting with filtration paper to remove excess water. Samples were fixed in 10% formalin for at least 24 hrs, then embedded in paraffin, sectioned at 5 µm, and stained with hematoxylin and eosin. All tissues were examined by 2 animal pathologists in a blinded fashion (LHR, V.M.D, BWS, D.V.M.) and confirmed by a pathologist (MMI, M.D., Ph.D.).

### Cell Culture

Cohorts of 3 wild-type and 3 EAF2^−/−^ C57BL/6J mice at 4 mos of age, and 5 wild-type and 5 EAF2^−/−^ FVB/NJ mice at 15 mos of age were euthanized and the anterior prostates were isolated for *in vitro* experiments. Anterior prostate lobes were microdissected from the urogenital tract and minced gently. Minced tissue was digested by 1 hr incubation at 37°C in 2.4 U/ml Dispase II (Roche Applied Science, Indianapolis, IN) in RPMI-1640 media on a rocker. The resultant cell suspension was aspirated with an 18-gauge needle and then cultured in DMEM with 10% FBS, 0.1 mmol/L nonessential amino acids (Invitrogen), 100 µmol/L 2-mercaptoethanol (Sigma), and penicillin/streptomycin. Anterior prostate epithelial and stromal cell mixtures were used at passage 1, subsequent passages 2–5 were used as enriched stromal cell populations. Lung stromal cells were isolated similarly for use as non-androgen-responsive control cells.

Growth curves were plotted to evaluate population growth of isolated stromal cells; characterization was performed at passages 2–5 in 12-well plates in triplicate, with seeding density of 1×10^5^ cells/well at day 0. Cells were harvested and counted with a haemocytometer on days 3, 5 and 9, and results plotted on a linear scale. The cell population doubling time was calculated during the logarithmic growth phase with Doubling Time Software v1.0.10 (Roth, 2006, http://www.doubling-time.com/compute.php). Each experiment was performed in triplicate and repeated a minimum of 3 times.

Human prostate carcinoma cell line C4-2, initially derived from a tumor xenograft induced by co-inoculating LNCaP prostate cancer sublines with bone stromal cells [16], was kindly provided by Dr. Leland W.K. Chung. Conditioned medium was obtained from wild-type and EAF2^−/−^ stromal cells at passages 2–4 cultured for 3–6 days to a confluency of ∼75%. C4-2 cells were plated at a density of 1×10^5^ cells/well in 12-well plates with 1 ml of stromal conditioned media from either wild-type or EAF2^−/−^ stromal cells at a confluency of ∼75%. Media was replaced with fresh stromal conditioned medium every 3 days. Cells were harvested and counted with a haemocytometer on days 2, 5 and 7. Results were plotted on a linear scale. Experiments were performed in triplicate a minimum of 3 separate times.

### In situ Hybridization

Before hybridization, murine prostate tissue cryosections (ProbeOn, Fisher Biotech, Pittsburgh, PA) were washed with PBS, fixed in 4% paraformaldehyde, digested with proteinase K at 20 µg/ml in PBS, refixed in 4% paraformaldehyde, rewashed in PBS, and then acetylated in 0.25% acetic anhydride in 0.1 M triethanolamine, pH 8.0. Full-length EAF2 cDNA was inserted into the EcoRI and XhoI site between T3 and T7 promoters in pBluescript II SK plasmid vector. The plasmid was purified by CsCl double banding, linearized with EcoRI or XhoI, and proteinase K-treated. Purified linear DNA templates were used in the synthesis of both sense and antisense digoxygenin-labeled riboprobes using either T3 or T7 RNA polymerase (Promega Corp., Madison, WI) as previously described [17], [18]. Riboprobe size was reduced to approximately 250 bp using limited alkaline hydrolysis.

For hybridization, the probe was diluted in hybridization solution (5×SSC, 1×Denhardt’s, 100 µg/ml salmon testis DNA, 50% formamide, and 250 µg/ml yeast transfer RNA), and slides were hybridized overnight at 67°C in a sealed chamber humidified with 5×SSC/50% formamide. Coverslips were removed, and slides were washed in 0.2×SSC at 72°C for 1 h. After washing in buffer (0.1 M Tris (pH 7.6), 0.15 M NaCl), slides were blocked in 10% horse serum at room temperature for 1 h. Slides were then incubated overnight at 4°C with antidigoxigenin-AP Fab fragments (1∶2000, Boehringer Mannheim, Mannheim, Germany) in 1% horse serum. Slides were washed and then developed with nitro blue tetrazolium (2.25 µl/ml) and 5-bromo-4-chloro-3-indolyl-phosphate, 4-toluidine salt (0.6 µg/ml) in alkaline phosphatase buffer (0.1 M Tris (pH 9.5), 0.05 M MgCl2, 0.1 M NaCl).

### Generation of EAF2 Monoclonal Antibody

GST-EAF2 fusion protein was generated by cloning human EAF2 cDNA into pGEX-2T vector (Amersham-Pharmacia, Piscataway, NJ). Female 6 week old BALB/c mice were immunized 9 times with GST-EAF2. Hybridomas were generated by fusing spleenocytes of an immunized mouse with mouse myeloma cells P3-X63-Ag8.653 [19]. Culture supernatants from antibody-secreting hybridoma cells were screened for specific reactivity with EAF2 transfected cells by Western blot and subcloned as described [20]. EAF2 mouse monoclonal antibodies were purified from ascites fluid as described [21]. The purity of EAF2 mouse monoclonal antibody preparations was assessed by SDS-PAGE and the activity by western blot.

### Human Prostate Tissue Specimens

Human prostate tissue specimens without any previous chemo-, radio- or hormone therapy were obtained from the surgical pathology archives by the University of Pittsburgh Prostate Tumor Bank from de-identified tumor specimens consented for research at time of treatment. Specimens included 14 matched prostate cancer and benign prostate specimens adjacent to prostate cancer. All cancer specimens were Gleason score ≥7. Use of these prostate tissues was approved by the University of Pittsburgh Institutional Review Board.

### Immunohistochemistry

Immunohistochemical stains were performed on five-micron sections of paraffin-embedded murine or human prostate tissue specimens as described previously [22], [23]. Briefly, sections were deparaffinized and rehydrated through a graded series of ethanol. Heat induced epitope retrieval was performed using a decloaker, followed by rinsing in TBS buffer for 5 minutes. Primary antibodies (working dilution 1∶400) rat monoclonal anti-CD31 (MEC 13.3, 550274, BD Biosciences, San Jose, CA, USA), rat monoclonal anti-Ki-67 (TEC-3, M7249, Dako, Carpinteria, CA, USA), rabbit polyclonal anti-tenascin-C (TNC) (AB19013, Chemicon, Billerica, MA, USA), goat polyclonal anti-ADAMTS-1 (L-18, sc-5486, Santa Cruz), and rabbit polyclonal caspase 3 (H-277, sc-7148, Santa Cruz Biotechnology) were used for immunostaining of murine tissue sections. Primary antibodies anti-CD34 (QBEnd/10, 790–2927, Ventana, Tucson, AZ, USA), rabbit polyclonal ADAMTS1 (NBP1-50161, Novus Biologicals, Littleton, CO, USA) and an internally generated mouse monoclonal anti-EAF2 were used for immunostaining of human prostate tissue specimens. Slides were then counterstained in hematoxylin and coverslipped. Immunostained sections were imaged with a Leica DM LB microscope (Leica Microsystems Inc, Bannockburn, IL, USA) equipped with a QImaging MicroPublisher 3.3 RTV digital camera (QImaging, Surry, BC, Canada).

For murine tissues, CD31-positive vessel density and Ki-67-positive cell density in were determined by analysis of sections from at least 3 independent mice from each genotype. Assessment of microvessel density was determined based on CD31-positive vessel count as described [9]; proliferative index was determined based on cell count as described [24]. Microvessel density for each group was determined from at least 20 fields imaged at 20X magnification with no overlap and identified by evaluating histological sections, and CD-31-positive vessels were counted to determine the average vessel numbers per field for each section. One section for each mouse was quantified. Proliferative index in mice on a C57BL/6J background was determined from at least 20 fields imaged at 20X magnification with no overlap. Ki-67-positive prostate epithelial cells were counted to determine the average number of proliferating cells for each section. For mice on an FVB/NJ background, slides stained with Ki-67 were scanned and digitized using the Aperio ScanScope CS scanner (Aperio, Vista, CA) to capture digital whole slide images (WSI) using the ×20 objective lens at a spatial sampling period of 0.47 µm per pixel. The digital WSI were analyzed using Aperio ImageScope software (http://www.aperio.com/pathology-services/image-scope-slide-viewing -software.asp). The manufacturer’s (Aperio Technologies, Inc.) algorithms were used to quantify nuclear staining.

For human prostate tissue specimens, CD34-positive vessel density was determined by analysis of prostate cancer specimens and matched benign prostate adjacent to prostate cancer specimens. Microvessel density was determined by counting the total number of CD34-positive vessels in at least 3 fields at 20X magnification. EAF2 and ADAMTS1 staining intensities were evaluated semi-quantitatively. The percentage of prostate epithelial cells of a specific histological phenotype (normal and cancer) that expressed the antigen was estimated in three randomly selected fields at a final magnification of 40X. Staining intensity was evaluated by two parameters (staining intensity and percentage of cells exhibiting each level of intensity). Intensity of reaction product was based on a 4-point scale – none, faint/equivocal, moderate and intense. An H-Score was calculated for each immunostain by cell type using the following formula:

H-Score = 0(% no stain) + 1(% faint/equivocal) + 2(% moderate) + 3(% intense). All tissues were examined by a pathologist in a blinded fashion (AVP, M.D., Ph.D.).

### Laser-capture Microdissection and Quantitative PCR

Murine samples were processed as described previously [25], using the Cells Direct™ OneStep qPCR kit from Invitrogen (Carlsbad, CA) according to manufacturer’s protocol including DNAse digestion step to ensure that starting material included no genomic DNA. Human prostate tumor specimens without any previous chemo-, radio- or hormone therapy were sectioned and evaluated by a board-certified pathologist (AJP). Prostate cancer cells and adjacent normal glandular cells were isolated by laser-capture microdissection using a Leica LMD6000 Microsystems microscope (Wetzlar, Germany) equipped with a HV-D20P Hitachi (Tokyo, Japan) color camera and Leica Laser Microdissection V 6.3 imaging software (Wetzlar, Germany). Primers were designed to span exon exon junctions using PrimerBank [26] or Primer3 software (Totowa, NJ,) and optimized to within 98–102% efficiency. Efficiencies were incorporated into calculations for qPCR using the ΔCp (crossing point) method (R = 2^[Cp sample – Cp control]^) in which the relative expression ratio (R) for each sample was to GAPDH. Primers used are listed in Table 1. All assays were run on an ABI Step-One Plus thermocycler (Applied Biosystems Inc., Carlsbad, CA).

**Table 1:** Primers for qPCR.

Gene	Forward	Reverse	Species
ADAMTS1	CATTAACGGACACCCTGCTT	CGTGGGACACACATTTCAAG	mouse
ADAMTS1	GCTCATCTGCCAAGCCAAAG	CTACAACCTTGGGCTGCAAAA	human
CD90	TGCTCTCAGTCTTGCAGGTG	TGGATGGAGTTATCCTTGGTGTT	mouse
EAF2	GGATTGGCATGTTTGCAGTTC	ACTGGATTCTGCTACTCCCTTC	mouse
EAF2	CCAGGACTCCCAATCTTGTAAA	TAGCTTCTGCCTTCAGTTCTCTT	human
GAPDH	AGGTCGGTGTGAACGGATTTG	GTAGACCATGTAGTTGAGGTCA	mouse
GAPDH	CATGTTCGTCATGGGTGTGA	GGTGCTAAGCAGTTGGTGGT	human
MMP9	CTGGACAGCCAGACACTAAAG	CTCGCGGCAAGTCTTCAGAG	mouse
OCN	GACCGAGTGCGGTTCAAAG	CGCAGGGCACATCCAACTT	mouse

### Statistical Analysis

Comparison between groups were calculated using the Student’s t-test, the two-tailed Fisher’s exact test method of summing small p values, the 1-way and 2-way ANOVA and Bonferonni’s Multiple Comparison Test as appropriate. Pearson correlation and Spearman Coefficient were calculated for immunostaining intensity of EAF2 and CD34-positive microvessel density in human prostate tissue specimens and categorized as follows: no correlation (0.00 to 0.30), moderate correlation (0.31 to 0.79), strong correlation (0.80 to 0.99). A value of p<0.05 was considered significant. GraphPad Prism version 4 was used for graphics (GraphPad Software, San Diego, CA, USA). Values are expressed as means ± S.E.M.

## Results

### EAF2-deficiency Induces a Reactive Stroma and PIN Lesions in Aged C57BL/6J and FVB/NJ Mice

**Figure 1:**
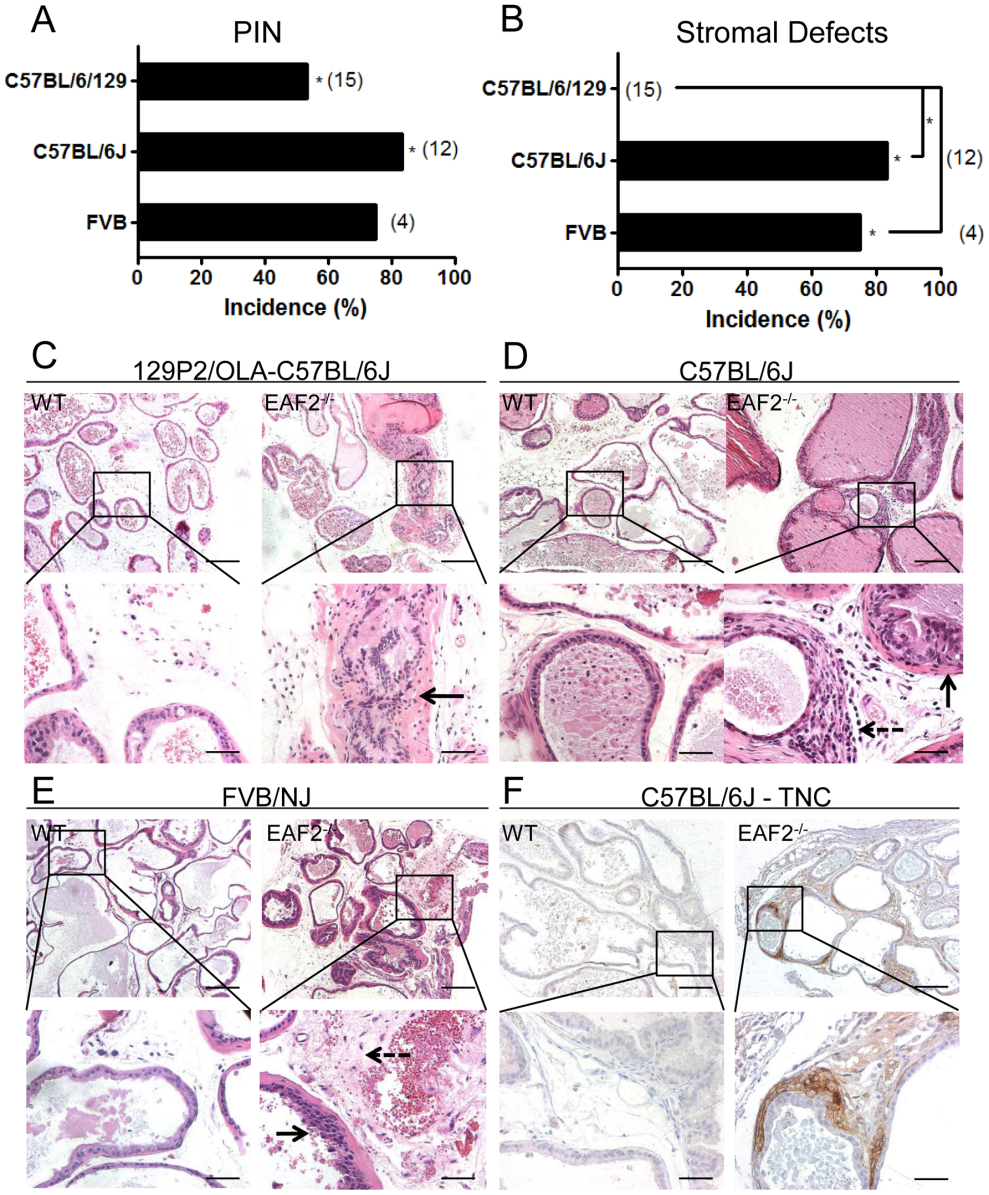
EAF2-deficiency induces murine prostatic intraepithelial neoplasia (mPIN) and stromal defects in C57BL/6J and FVB/NJ mice at age 20–24 mos. A. Strain-specific incidence rate of mPIN in EAF2^−/−^ mice at age 20–24 mos compared to 0% incidence in wild-type (WT) controls (*p<0.01). B. Strain-specific incidence in stromal defects in EAF2^−/−^ mice at age 20–24 mos compared to 0% incidence in WT controls (*p<0.01). Number of animals in each group is indicated in parenthesis. C. EAF2^−/−^ mice on a 129P2/OLA-C57BL/6J background developed mPIN lesions but not stromal defects (right panel inset, black arrow designating mPIN). D. Aged EAF2^−/−^ mice on a C57BL/6J background displayed mPIN and stromal hypertrophy (right panel, solid black arrow) and interstitial fibrosis (right panel inset, dashed arrow) at age 20–24 mos compared to WT controls. E. Aged EAF2^−/−^ mice on an FVB/NJ background displayed mPIN (right panel inset, solid black arrow) and increased stromal inflammation and fibrosis (right panel inset, dashed black arrow) compared to WT controls. F. Prostate stromal cells surrounding mPIN lesions in aged EAF2^−/−^ mice displayed immunopositivity for reactive stroma marker tenascin-C (TNC). Original magnification 10X, inset 40X. Scale bars indicate 200 micron in 10X, 50 micron in 40X. All images are ventral prostate lobe.

EAF2^−/−^ mice were generated on a pure C57BL/6J or a pure FVB/NJ background in order to determine the potential effects of different genetic backgrounds on the prostate phenotype. All genetic backgrounds examined had a statistically significant increased incidence in mPIN lesions in EAF2^−/−^ animals compared to wild-type controls at 20–24 mos of age (Fig. 1A). On a 129P2/OLA-C57BL/6J genetic background, mPIN lesions were reported in 8 out of 15 (53%) EAF2^−/−^ animals at age 24 mos [8]. Similarly, EAF2^−/−^ mice on a pure C57BL6/J background developed mPIN lesions in 10 out of 12 (83.3%, p<0.05) and in 3 out of 4 (75%, p = 0.1429) FVB/NJ animals at age 20–24 mos compared to wild-type controls (Fig. 1A). There was no statistically significant difference in mPIN incidence among the strains. No wild-type animals displayed mPIN lesions in any strain. One striking difference apparently influenced by genetic background was the lack of macroscopic tumors in other organs of aged mice on a C57BL/6J and FVB/NJ background, suggesting that genetic background has a significant impact on carcinogenesis induced by EAF2 loss. Another difference was in the incidence of prostate stromal abnormalities in aged C57BL/6J and FVB/NJ EAF2^−/−^ animals associated with PIN lesions that were characteristic of a reactive stroma (Fig. 1B). Prostate reactive stroma is characterized by decreased smooth muscle differentiation, increased myofibroblasts and fibroblasts and an increase in extracellular matrix remodeling [3]. Stromal defects were not previously reported in EAF2^−/−^ mice on a 129P2/OLA-C57BL/6J background [8]. Re-examination of the prostates from 129P2/OLA-C57BL/6J background mice by a board certified animal pathologist (L.H.R.) verified the lack of stromal abnormalities in either EAF2^−/−^ or wild-type animals on a 129P2/OLA-C57BL/6J background (Fig. 1C). However, all of the C57BL6/J (10/12) and FVB/NJ (3/4) EAF2^−/−^ mice displaying mPIN lesions at age 20–24 mos also displayed stromal abnormalities when compared to wild-type controls (Fig. 1D, E). The mPIN lesions in EAF2^−/−^ animals on a pure C57BL/6J or FVB/NJ background were characterized by increased stromal hypertrophy and hyperplasia, increased lymphatic dilation and chronic inflammation compared to age-matched wild-type controls (Fig. 1 D, E). Furthermore, EAF2^−/−^ animals on a C57BL/6J background displayed areas of moderate to intense stromal immunopositivity for reactive stroma marker tenascin-C (TNC) [3], [27], whereas wild-type animals displayed no immunopositivity for TNC expression (Fig. 1F). Age-related mild multifocal lymphocytic interstitial infiltrates were observed in all of the C57BL/6J and FVB/NJ wild-type and EAF2^−/−^ prostates of mice at 20–24 mos (data not shown). The development of a reactive stroma in the C57BL6/J and FVB/NJ but not the 129P2/OLA-C57BL/6J suggests that genetic background influences the EAF2 knockout prostate phenotype.

### Temporal Development of Prostate Stromal and Epithelial Defects in EAF2-Deficient Mice

**Figure 2:**
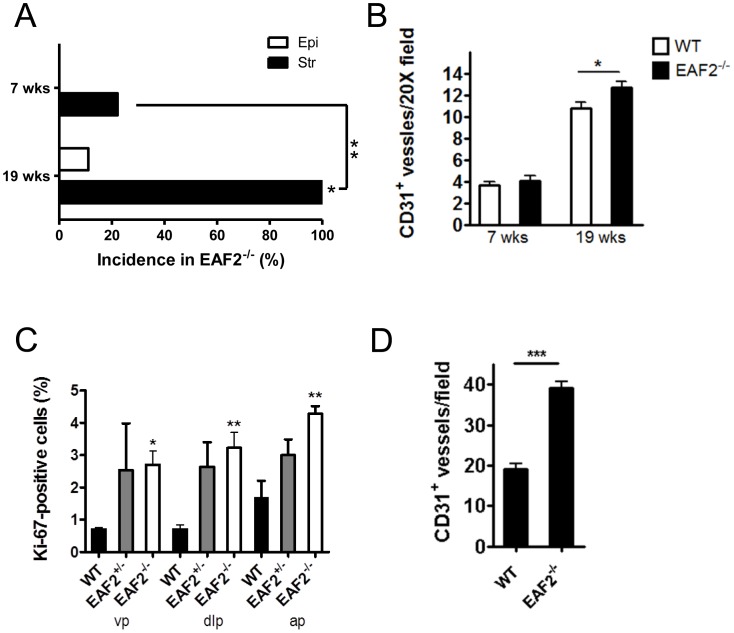
Effects of EAF2 loss on C57BL/6J and FVB/NJ mouse prostate. A. Incidence in prostate epithelial hyperplasia (Epi) and stromal defects (Str) in C57BL/6J EAF2^−/−^ mice at age 7 wks and 19 wks compared to 0% incidence in age-matched wild-type controls. B. CD31^+^ microvessel density in C57BL/6J wild-type (WT) and EAF2^−/−^ mice at age 7 wks and 19 wks. Data represent average of 9 C57BL/6J mice per group. C. Percentage of Ki-67 positive epithelial cells in the ventral (vp), dorsal-lateral (dlp), and anterior (ap) prostate of wild-type (WT), EAF2^+/−^ and EAF2^−/−^ FVB/NJ mice at age 20 mos. D. CD31^+^ microvessel density in FVB/NJ WT and EAF2−/− mice at age 20 mos. Data represent average of 3 FVB/NJ mice per group. (*p<0.05, **p<0.001, ***p<0.0001).

In order to further characterize the effects of EAF2 loss on the development of prostate epithelial defects and the development of a reactive stroma, the prostates of C57BL/6J wild-type and EAF2^−/−^ mice were examined at age 7 weeks, and 19 weeks (n = 9 per group). Prostate abnormalities including epithelial hypertrophy, hyperplasia and dysplasia were previously reported in EAF2^−/−^ mice on a 129P2/OLA-C57BL/6J genetic background as early as 4–6 months of age [8]. On a C57BL/6J background at age 7 weeks, no histological epithelial abnormalities were detected in wild-type or EAF2^−/−^ animals (Fig. 2A and Fig. S1A). Two EAF2^−/−^ animals had mild smooth muscle proliferation, fibroplasia and lymphocytic infiltration compared to wild-type animals (p = 0.47). By 19 weeks of age, 1 out of 9 EAF2^−/−^ mice on a C57BL/6J background displayed small areas of prostate epithelial hyperplasia (Fig. S1B, solid black arrow). However 9 out of 9 (100%, p<0.0001) EAF2^−/−^ mice displayed diffuse, evenly distributed mild stromal inflammation (Fig. 2A and Fig. S1B, dashed arrow). Compared to the wild-type prostate stroma, EAF2^−/−^ animals at age 19 weeks had increased edema, lymphocytic infiltration, plasma cells, neutrophils, macrophages, mast cells and fibroblasts. Microvessels in the prostates of EAF2^−/−^ mice were frequently associated with inflammation (Fig. S1B, black arrowhead). The prostates of both wild-type and EAF2^−/−^ mice displayed no immunopositivity for reactive stromal marker TNC at age 19 weeks (data not shown).

### EAF2 Deficient Mice have Increased Prostate Microvessel Density

In order to further characterize the potential contribution of increased microvessel density and cellular proliferation to the development of epithelial defects in the EAF2-deficient murine prostate, the number of CD31 positive vessels and Ki-67 positive epithelial cells was quantified in wild-type and EAF2^−/−^ animals at ages 7 and 19 weeks on a pure C57BL/6J background. In agreement with the lack of histological epithelial abnormalities in EAF2^−/−^ mice at ages 7 and 19 weeks (see Fig. 2A), there was no significant difference in cellular proliferation between EAF2^−/−^ and wild-type control animals (data not shown). Previously, the prostates of EAF2 knockout mice on the C57BL/6J background had statistically significant decreased expression of anti-angiogenic TSP-1 at 3 mos of age [10], and an approximate 2-fold increase in microvessel density age 20–24 mos [9]. While there was no statistically significant difference in microvessel density detected at age 7 weeks, a small but statistically significant increase in the number of CD31-positive vessels per field was observed in the prostates of 19-week old EAF2^−/−^ mice as compared to wild-type control animals (Fig. 2B and Fig. S1C). In agreement with increased prostate epithelial cell proliferation and microvessel density in aged mice on the C57BL/6J background [9], EAF2^−/−^ FVB/NJ mice at 20 mos of age had a statistically significant increase in Ki-67 positive cells and a statistically significant increase in CD31^+^ vessels compared to age-matched wild-type controls (Fig. 2C, D and Fig. S1D, E). Although not statistically significant, EAF2^+/−^ FVB/NJ animals at 20 mos of age also had an increased cellular proliferation, suggesting that heterozygous loss of EAF2 could also contribute to the development of epithelial defects. These results suggest that microvessel proliferation and stromal inflammation could precede the development of prostate epithelial abnormalities such as hyperplasia and PIN lesions. As there was not a statistically significant increase in the incidence in PIN lesions in the C57BL/6J (83.3%) and FVB/NJ EAF2 (75%) knockout models compared to that of the 129P2/OLA-C57BL/6J (53%), perhaps due to an insufficient sample size to determine this endpoint, it is not clear whether the stromal defects contributed to the development of PIN lesions in these mice. However, EAF2 knockout was associated with an increased incidence in murine PIN lesions in all three of these strains.

**Figure 3:**
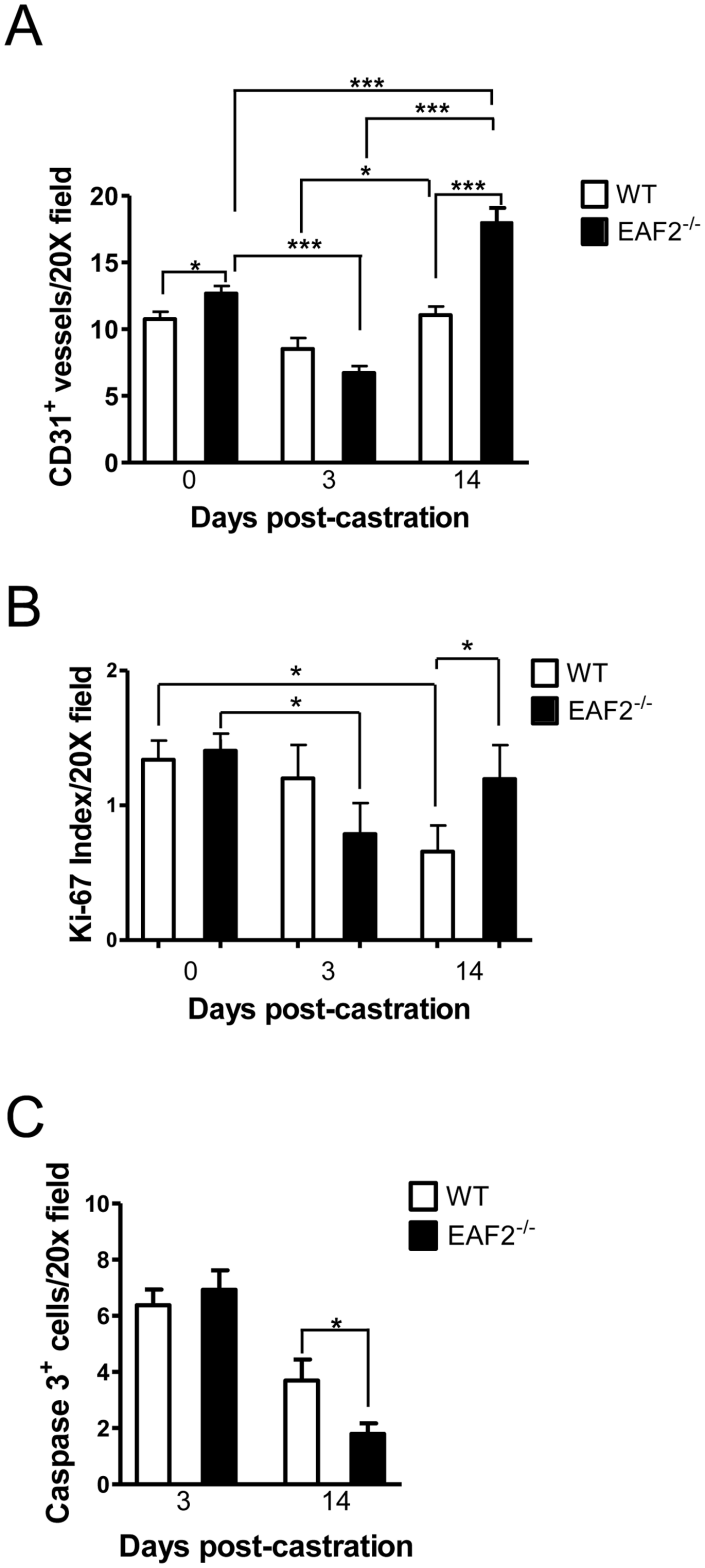
Effects of castration on EAF2-deficient mice at age 19 wks. A. Quantification of CD31^+^ vessels in intact (0 days) and castrated EAF2^−/−^ mice vs wild-type (WT) controls on Day 3 and Day 14 post-castration. B. Quantification of Ki-67^+^ cells in intact and castrated EAF2^−/−^ mice vs WT controls on Day 3 and Day 14 post-castration. C. Quantification of caspase 3^+^ luminal epithelial cells in castrated EAF2^−/−^ mice vs WT controls on Day 3 and Day 14 post-castration. Data represent average of at least 3 mice per group. (*p<0.05, ***p<0.001).

Androgens regulate prostate growth in part through effects on the vascular microenvironment [28], [29]. In order to determine the effects of androgen deprivation on prostate neovascularization, cellular proliferation and apoptosis in the EAF2 knockout, mice were castrated at age 19 weeks and euthanized at 3 and 14-days post-castration. Human prostate cancer xenografts have been shown to recover completely from vascular damage due to androgen deprivation within 5–7 days [30]. In agreement with this finding, wild-type and EAF2^−/−^ animals had decreased prostate vascularity at 3 days post-castration (Fig. 3A). At 14 days post-castration, microvessel density had recovered in both wild-type and EAF2^−/−^ animals, suggesting that the murine prostate vasculature responds similarly to xenograft tumors in response to androgen deprivation (Fig. 3A and Fig. S2A). Compared to wild-type controls, EAF2^−/−^ mice had a decreased microvessel density at 3 days post-castration and an increased microvessel density at 14 days post-castration. Furthermore, this fluctuation in vascularity was statistically significant at both time points. At 3 days post-castration, EAF2^−/−^ was decreased 1.89-fold (p<0.001) and increased 1.42-fold (p<0.01) compared to the microvessel density of intact EAF2^−/−^ mice. These results suggest that EAF2 plays a significant role in the maintenance of the prostate vasculature even in the absence of androgens.

In response to castration, the prostates of both wild-type and EAF2^−/−^ animals were involuted by day 14 and significantly decreased in mass compared to intact controls. However, EAF2^−/−^ mice had a statistically significant increase in cell proliferation as shown by an elevated number of Ki-67 positive cells (Fig. 3B and Fig. S2B) and a statistically significant decrease in apoptosis as shown by a decreased number of caspase-3-positive cells compared to castrated wild-type at day 14 (Fig. 3C and Fig. S2C). Although not statistically significant compared to wild-type controls, EAF2^−/−^ animals had a statistically significant response in reduced vascularity and cellular proliferation at day 3 post-castration compared to intact EAF2^−/−^ animals. At day 14 post-castration, the increased microvessel density, cellular proliferation and reduced apoptosis suggested that EAF2^−/−^ mice were less responsive to castration-induced prostate involution. Taken together, these results suggest that EAF2 loss may be associated with an increased recovery rate and a decreased overall response to the effects of androgen deprivation in the murine prostate.

### EAF2 Expression is Localized to the Epithelial Cell in Murine Prostate

**Figure 4:**
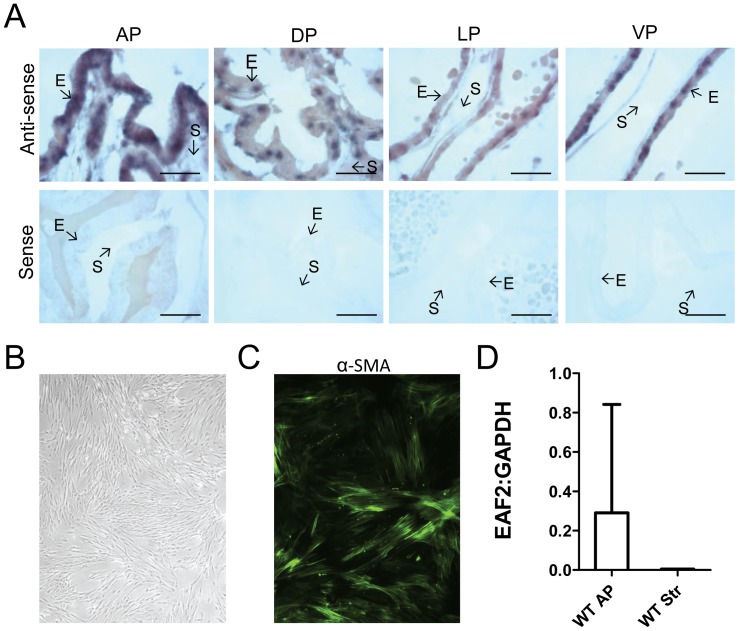
Expression of EAF2 in the murine prostate. A. In situ hybridization analysis of EAF2 mRNA in murine prostate tissue. Both anti-sense (upper panels) and sense (lower panels) EAF2 RNA probes were labeled with DIG and visualized with alkaline phosphatase-conjugated anti-DIG antibody. Epithelial (E) and stromal (S) cells are indicated by arrows. AP, anterior prostate, DP, dorsal prostate, LP, lateral prostate, VP, ventral prostate. Scale bars indicate 50 micron. B. Stromal cells isolated from the anterior prostate cultured *in vitro* at passage 3. C. Stromal cells express α-smooth muscle actin (α-SMA). D. qPCR analysis of EAF2 expression in cultured mixed epithelial and stromal cells isolated and from wild-type AP (WT AP) compared to wild-type stromal cells (WT Str). Data are normalized to GAPDH. Data represent average of 3 animals per cohort.

In human prostate tissue, EAF2 protein expression was previously reported as confined to the epithelial cell [6]. In situ hybridization was used to determine the cell type specific expression of EAF2 mRNA in the wild-type murine prostate. EAF2 mRNA expression was localized to prostate epithelial cells with little to no expression in the stromal cells (Fig. 4A). In addition, anterior prostate lobes were isolated from wild-type mice at age 15 mos and cultured *in vitro.* Mixed prostate epithelial and stromal cells were analyzed by qPCR at passage 1 and compared to enriched stromal cell populations cultured *in vitro* for 2–4 passages by qPCR. Enriched stromal cells displayed the elongated spindle-shaped morphology typical of cultured fibroblasts and expressed stromal marker α-smooth muscle actin (αSMA) (Fig. 4B, C). Analysis of EAF2 mRNA in the anterior prostate passage 1 mixed stromal and epithelial cell cultures compared to the passage 2–4 enriched stromal cell cultures demonstrated a decrease in EAF2 expression in the stromal cells (Fig. 4D). These results suggest that, in agreement with EAF2 expression in the human prostate, EAF2 expression in the murine prostate is predominantly localized to epithelial cells.

**Figure 5:**
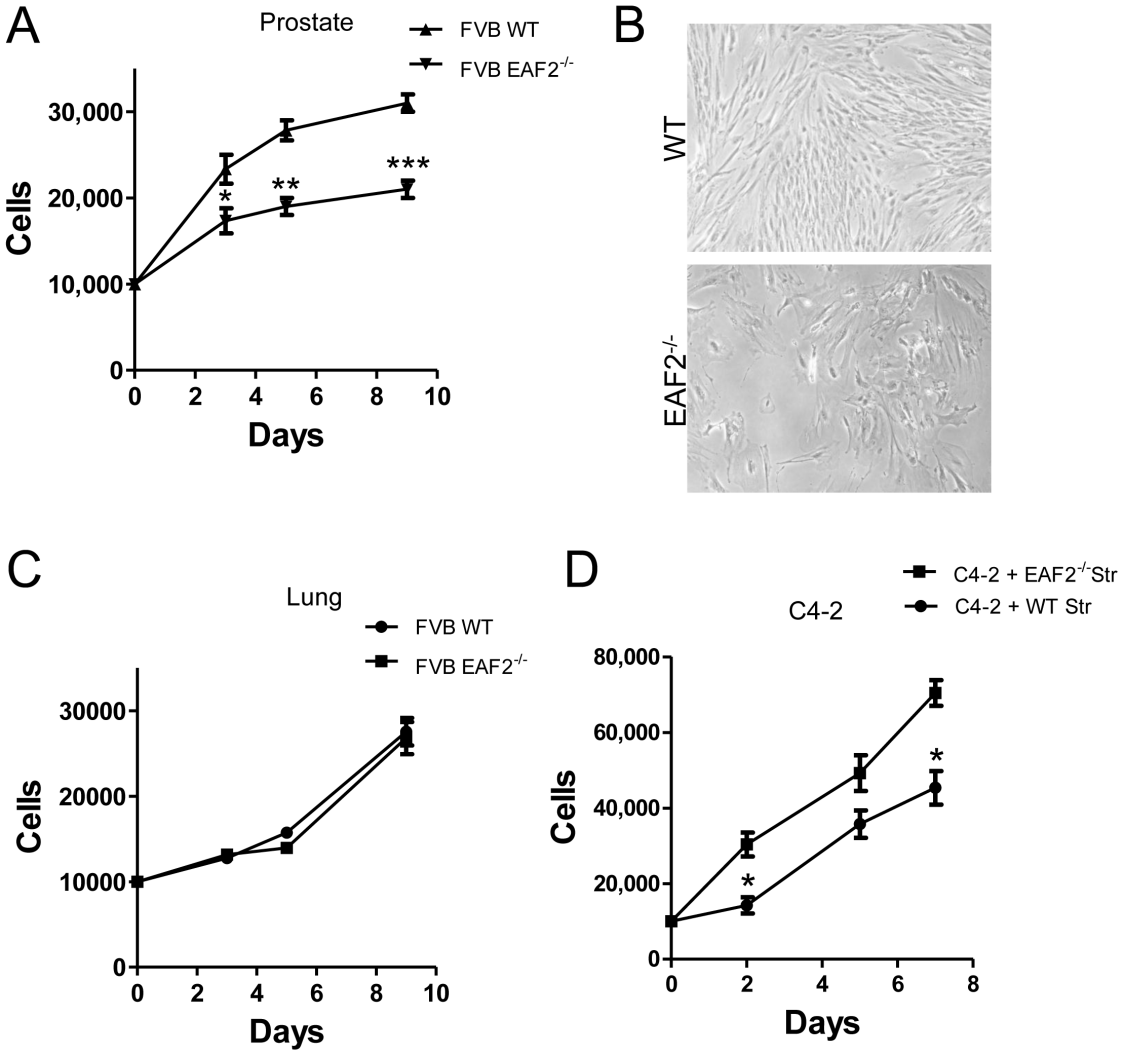
Growth characteristics of stromal cells isolated from wild-type and EAF2^−/−^ mice. A. Growth curve analysis (in days) of cultured prostate stromal cells isolated from wild-type (WT) and EAF2^−/−^ FVB/NJ mice at age 15 mos. B. Stromal cells cultured in vitro from WT and EAF2^−/−^ mice. C. Growth curve analysis of cultured lung stromal cells isolated from WT and EAF2^−/−^ FVB/NJ mice at age 15 mos. D. Growth curve analysis of C4-2 cells grown in prostate stromal cell-conditioned media from WT and EAF2^−/−^ FVB/NJ mice at age 15 mos. Data represent average a minimum of 3 animals per cohort and at least 3 technical replicates representative of 3 different experiments (*p<0.05, **p<0.001, ***p<0.0001).

In order to further characterize the impact of EAF2 loss on the prostate microenvironment, stromal cells were isolated and cultured *in vitro* from the prostates of EAF2^−/−^ and wild-type C57BL/6J mice at age 19 weeks and C57BL/6J and FVB/NJ mice at age 15 mos for 2–4 passages. Growth curves were not statistically different in EAF2^−/−^ C57BL/6J mice compared to wild-type controls at age 19 weeks (data not shown), however EAF2^−/−^ FVB/NJ mice at 15 mos of age demonstrated a statistically significant decreased growth rate compared to age-matched wild-type controls (Fig. 5A, B). Similar decreased growth rate was demonstrated by EAF2^−/−^ C57BL/6J mice at 15 mos of age (data not shown). Stromal cells isolated from both wild-type and EAF2^−/−^ mice from both strains became quiescent after passage 4. In addition to prostate, EAF2 expression has been identified in several adult murine tissues, including the adult brain, spleen, liver, lung, thymus, and kidney [31]. Growth curves of lung stromal cells were not statistically different in EAF2^−/−^ compared to wild-type FVB/NJ mice at 15 mos of age (Fig. 5C), suggesting the growth difference in stromal cells isolated from the EAF2 knockout was prostate specific. Initiated human prostate epithelial cells have an increased growth rate when co-cultured with cancer associated fibroblasts compared to normal prostate stromal cells [32]. Similarly, conditioned media from prostate stromal cells isolated from aged EAF2^−/−^ FVB/NJ mice induced a statistically significant increased growth rate in C4-2 prostate cancer cells compared to conditioned media from wild-type prostate stromal cells, suggesting that the altered stromal microenvironment induced by EAF2 loss in aged animals could stimulate epithelial cell growth through paracrine signaling (Fig. 5D).

### Decreased Expression of ADAMTS1 in EAF2^−/−^ Mice

Since EAF2 in murine prostate is expressed by epithelial cells (see Fig. 5), signals inducing the modified EAF2^−/−^ prostate stromal and vascular microenvironment could be paracrine. The prostate stromal microenvironment has been postulated to limit tumor growth through the up-regulation of genes inhibiting proliferation and angiogenesis, including ADAMTS1 [33]. ADAMTS1 is a secreted protein that acts as an inhibitor of angiogenesis, invasion and metastasis. Normal prostate stromal cells isolated from human tissue specimens have been shown to induce the up-regulation of several genes encoding secreted proteins including ADAM metallopeptidase with thrombospondin (TSP) type I motif, 1 (ADAMTS1) whereas CD90^+^ cancer associated stromal fibroblasts did not [34]. Furthermore, Altered ADAMTS1 expression was not identified as differentially expressed in microarray analyses of young EAF2^−/−^ mice [35] or in qPCR analyses of murine prostate at age 19 weeks (data not shown), suggesting that EAF2 does not directly regulate ADAMTS1 expression. Although not statistically significant, analysis of FVB/NJ murine prostate stromal cells isolated from mice at 15 mos of age revealed an increase in mRNA expression in CD90 (p = 0.096) and a decrease in ADAMTS1 (p = 0.0582) in EAF2^−/−^ mice compared to wild-type controls (data not shown). Genetic background can have a significant impact on prostate tumor development in mice [36], [37], and mPIN lesions were more frequently observed in C57BL/6 and FVB/N strains than in the 129/SvImJ background (reported in [38]). In an analysis of prostate transcriptomes from multiple genetic strains, ADAMTS1 was among the most differentially expressed [38]. Baseline differences in the expression of genes that can either contribute to or inhibit tumor initiation and progression can have a significant impact on knockout phenotype. The development of a reactive stroma in the EAF2^−/−^ mice on the C57BL/6J and FVB/NJ genetic backgrounds but not 129P2/OLA-C57BL/6J could be due in part to secondary strain-specific differences that exacerbate the effects of EAF2 loss in the prostate microenvironment.

### EAF2 Expression is Negatively Correlated with Microvessel Density in Human Prostate Cancer Tissue Specimens

**Figure 6:**
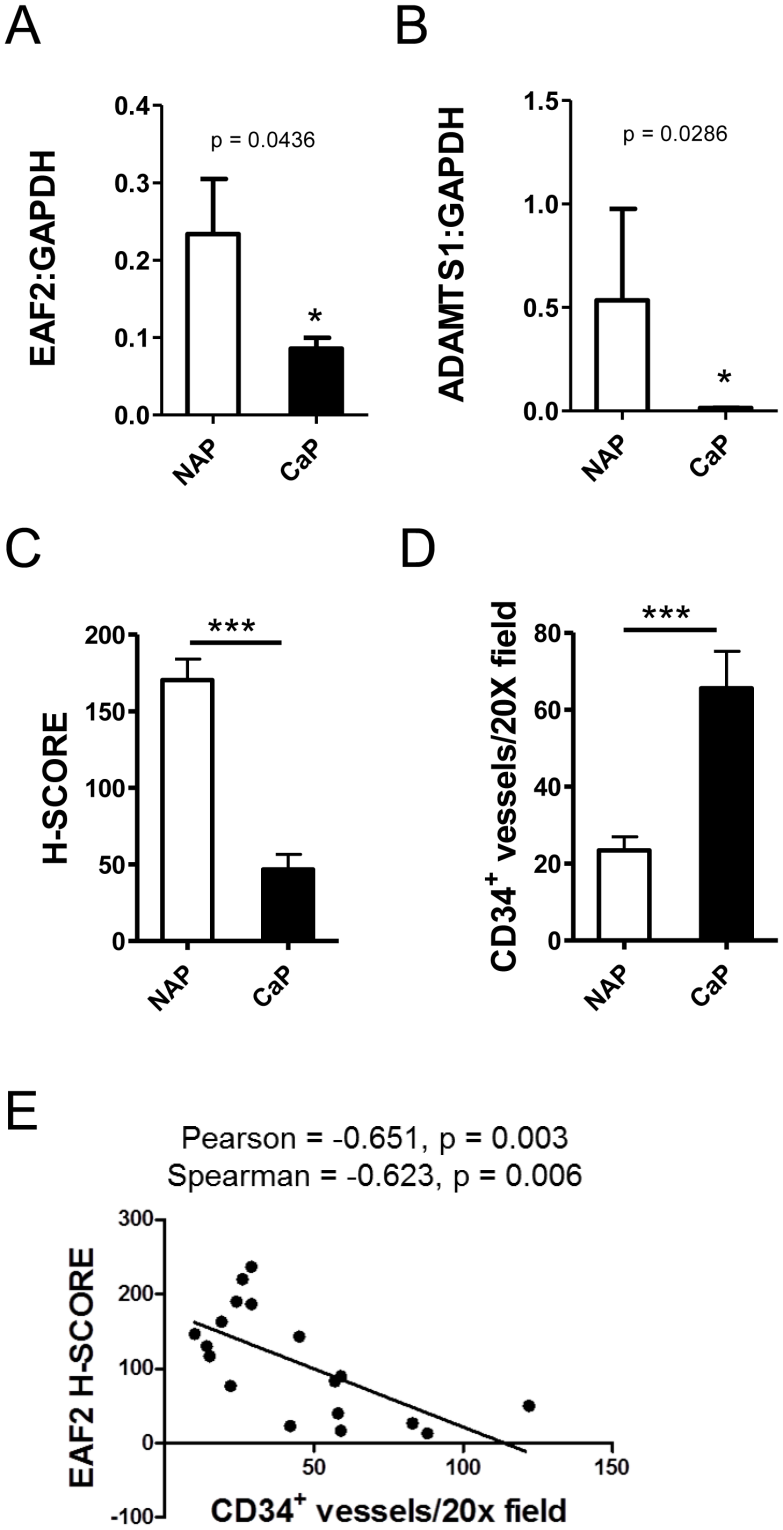
Correlation of EAF2 down-regulation and vascularity in human prostate cancer tissue specimens. A. qPCR analysis of EAF2 expression in human prostate cancer tissue specimens (CaP) and normal adjacent prostate tissues (NAP). B. ADAMTS1 expression in CaP and NAP. qPCR data represent 6 patients (*p<0.05), normalized to GAPDH. C. Quantification of EAF2 staining intensity H-Score in matched NAP and prostate cancer (CaP) tissue specimens. D. Quantification of CD34-positive vessels per 20X field from matched NAP and CaP specimens. E. Scatter plot of EAF2 immunostaining intensity and CD34-positive microvessel density in human prostate tissue specimens. Statistical Pearson correlation was −0.651 (p = 0.003) and Spearman Coefficient was −0.623 (p = 0.006). EAF2 immunostaining intensity was negatively correlated with CD34-positive microvessel density in human prostate tissue specimens. Immunostaining data represent 9 patients. (***p<0.0001).

EAF2 has previously been shown to be down-regulated in human prostate cancer specimens [6]. ADAMTS1 expression is also decreased in human prostate cancer [39]. In order to further examine the potential correlation of EAF2 and ADAMTS1 in human prostate cancer, mRNA levels of EAF2 and ADAMTS1 were analyzed in laser capture microdissected human prostate tissue specimens (Fig. 6A, B and Fig. S3A). Additionally, the expression intensity of EAF2, ADAMTS1 and the number of CD34-positive microvessels was also examined in human prostate tissue specimens by immunostaining (Fig. S3B). Both EAF2 and ADAMTS1 mRNA expression levels were significantly reduced in prostate cancer tissues compared to normal adjacent tissue (see Fig. 6A, B). Expression levels of EAF2 did not correlate with ADAMTS1 expression levels (data not shown) further suggesting that EAF2 does not directly regulate ADAMTS1 mRNA expression in the prostate.

Semi-quantitative analysis of immunostaining intensity revealed a negative correlation between EAF2 and the number of CD34-positive vessels in human prostate specimens. Compared to matched benign tissues, EAF2 staining intensity was significantly reduced in prostate cancer tissues while microvessel density was increased (Fig. 6C, D and Fig. S3). The Pearson correlation was −0.651 (p = 0.003) and Spearman Coefficient was −0.623 (p = 0.006) indicating a moderate negative correlation between EAF2 expression and CD34-positive microvessel density (Fig. 6E). The negative correlation of EAF2 expression and microvessel density in human prostate tissue specimens was in agreement with the increased vascularity observed in the EAF2 knockout mice, suggesting that EAF2 plays an important role in the regulation of prostate vascularity in both normal and malignant tissues.

## Discussion

Prostate stromal-epithelial interactions are critical for both development and maintenance of prostate homeostasis. Although the specific mechanisms that induce the development of a cancer-associated or “reactive” stroma are not fully understood, alterations in the prostate stroma have been associated with mPIN lesions in mice [40], and the development of a reactive stroma has been associated with the development of early PIN as well as cancer in human prostate [3], [41]. The presence of a reactive stroma in prostate cancer has been associated with biochemical recurrence, and has been also correlated with risk of prostate cancer specific death [42], [43]. In the current study, EAF2-deficiency in mice induced an increased incidence in prostatic intraepithelial neoplasia in a panel of murine strains. In addition to mPIN lesions, aged EAF2^−/−^ C57BL/6J and FVB/NJ mice also developed a statistically significant increase in prostate stromal defects characteristic of a reactive stroma, including hyperplasia, fibroplasia, increased microvessel density and increased expression of reactive and cancer-associated fibroblast stroma markers TNC. Mild prostate stromal inflammation was displayed by EAF2^−/−^ animals as early as 19 weeks of age and preceded the development of the increased epithelial cell proliferation displayed by older EAF2^−/−^ animals.

Previously, EAF2^−/−^ mice on a mixed 129P2/OLA-C57BL/6J genetic background showed significantly enhanced neovascularization in Matrigel plug angiogenesis assays [44]. Furthermore, male mice on a C57BL/6J background at 20–24 months of age displayed a statistically significant increase in microvessel density in the prostate [9]. In agreement with these previous studies, the EAF2 knockout model exhibited significantly increased prostate microvessel density in aged animals from the FVB/NJ strain. Previous studies have demonstrated that EAF2 loss in C57BL/6J mice was associated with a significant decrease in the expression of anti-angiogenic TSP-1 as early as 3 mos of age [10]. EAF2 knockout mice have also been shown to have increased levels of HIF1α at 12 mos of age [9]. Indeed, by 19 weeks of age, C57BL/6J mice displayed a statistically significant increased prostate vascularization in EAF2-deficient animals, and this increased vascularity appeared to precede the development of prostate epithelial hyperplasia and mPIN lesions in the aged EAF2 knockout model. Furthermore, microvessel density and cellular proliferation was also increased in the prostates of castrated EAF2^−/−^ animals compared to wild-type controls, suggesting that the EAF2^−/−^ prostate was less responsive to castration than wild-type.

Stromal influence on prostate tumor development and progression is not completely understood. Previous studies have demonstrated that stromal cells are capable of regulating angiogenesis in prostate tumors [45]. However, it is clear that the luminal epithelial cell also plays an important role. Similar to human prostate, EAF2 expression is confined to the epithelial cell in the murine prostate. In the current study, EAF2 loss was associated with increased incidence in mPIN lesions and increased vascularity in 3 murine strains and EAF2 down-regulation was associated with increased vascularity in human prostate tumors specimens. Furthermore, the EAF2^−/−^ murine prostate vasculature and epithelial cell proliferation responded to and recovered from castration more quickly than wild-type prostate. Recently, we reported that EAF2 down-regulation was more frequently observed in tumors with Gleason scores 8–9 than in Gleason score of 7 or lower [46]. Prostate tumor angiogenesis and cell proliferation are also positively correlated with Gleason score [47], [48]. These observations suggest that EAF2 can modulate prostate vascularity and epithelial cell proliferation, and loss of EAF2 in prostate tumors might contribute to progression and castration resistance.

On the C57BL/6J and FVB/NJ backgrounds, aged EAF2^−/−^ mice displayed increased incidence in mPIN lesions compared to wild-type controls as well as stromal defects characteristic of cancer-associated fibroblasts. In human prostate, it has been hypothesized that increasing cellular senescence in the aging prostate could induce remodeling of the prostatic stroma towards a more reactive phenotype [49]. Prostate stromal cells isolated from EAF2^−/−^ prostates and grown in culture had a statistically significant decreased growth rate compared to age-matched wild-type controls, whereas the growth rate of lung stromal cells was not altered. Prostate cancer-associated fibroblasts isolated from human tissues stimulate epithelial cell proliferation, whereas normal prostate stromal cells inhibit epithelial proliferation [32]. Similarly, EAF2^−/−^ prostate stromal cells also induced an increased growth rate in C4-2 cells compared to wild-type stromal cells. This finding suggests that reactive stroma in the EAF2 knockout mouse is functionally important and could promote prostate carcinogenesis. EAF2 knockout mice on the C57BL/6J or FVB/NJ background provide an in vivo model to study reactive stroma in early prostate carcinogenesis.

Prostate cancer-associated fibroblasts in human tissues have been identified by strong CD90 immunostaining [50]. In a co-culture assay, CD90^+^ prostate cancer-associated fibroblasts had a significantly decreased inductive signaling capability compared to normal prostate stromal cells [34]. Genes encoding secreted proteins ADAMTS1, IGFBP5 and WNT5A were significantly down-regulated in NCCIT cells co-cultured with CD90^+^ prostate cancer-associated fibroblasts compared to induction by normal prostate stromal cells [34]. The prostates of EAF2^−/−^ mice at 15 mos of age had decreased expression of ADAMTS1 and increased CD90 expression. EAF2 and ADAMTS1 expression were also decreased significantly in human prostate cancer compared to normal adjacent tissues. EAF2^−/−^ animals at age 19 weeks displayed evidence of stromal inflammation, but did not express reactive stroma marker TNC or decreased expression of ADAMTS1, suggesting that the reactive stroma and down-regulation of ADAMTS1 in EAF2^−/−^ murine prostate was a secondary effect that developed with age. Since ADAMTS1 was not down-regulated in EAF2^−/−^ mice at age 3 mos, it is likely that EAF2 does not directly affect ADAMTS1 expression and that the reactive stroma in the C57BL/6J and FVB/NJ EAF2 knockout model is strain-specific. EAF2 expression was negatively correlated with CD34-positive vessel density, supporting the findings in the murine model that implicate an important role for EAF2 in regulating prostate vascularity.

In summary, EAF2-deficient male mice on a pure C57BL/6J and pure FVB/NJ background displayed a statistically significant increase in PIN lesions similar to that found previously in mice on a 129P2/OLA-C57BL/6J background. In contrast to the 129P2/OLA-C57BL/6J genetic background, tumor incidence in other major organs was significantly reduced in EAF2^−/−^ mice on C57BL/6J or FVB/NJ backgrounds. The mPIN lesions in aged C57BL/6J and FVB/NJ EAF2^−/−^ mice were also characterized by a reactive stroma. C57BL/6J mice at 19 weeks of age exhibited stromal inflammation and increased microvessel density which preceded the development of PIN lesions and a reactive stroma. These pathologic changes could contribute to the development of PIN lesions and an accompanying reactive stroma in aged EAF2^−/−^ animals. In human prostate tissues, EAF2 expression was positively correlated with ADAMTS1 gene expression levels and negatively correlated with microvessel density, consistent with the results observed in the EAF2 knockout mouse model. Future studies will focus on elucidating the relationship between EAF2, ADAMTS1 and the stromal microenvironment in prostate tumor development and progression.

## Supporting Information

Figure S1 **Effects of EAF2 loss on C57BL/6J and FVB/NJ mouse prostate histology.** A. EAF2-deficiency induced stromal inflammation occurred as early as 7 wks in C57BL/6J mice. B. EAF2^−/−^ murine prostates displayed prostate epithelial hyperplasia (black arrow, inset), evenly distributed mild stromal inflammation characterized by increased edema, lymphocytic infiltration, plasma cells, neutrophils, macrophages, mast cells and fibroblasts (dashed arrow, inset) compared to wild-type (WT) at age 19 wks. Microvessels (black arrowhead, inset) in the prostates of EAF2^−/−^ mice were frequently associated with inflammation (black arrowhead, inset). Original magnification for A and B: 10X, inset 40X. Scale bars indicate 200 micron in 10X, 50 micron in 40X. C. CD31 immunostaining of microvessels (black arrowheads) in transverse sections of dorsal-lateral prostate lobes from wild-type (WT) control and EAF2^−/−^ mice on a C57BL/6J background at 19 weeks of age. Original magnification for C: 10X, inset 20X. Scale bars indicate 200 micron. D. Ki-67 immunostaining (black arrowheads) in transverse sections of prostate ventral (vp) and anterior (ap) lobes from WT and EAF2^−/−^ FVB/NJ mice at 20 mos of age. Original magnification 20X. Scale bar represents 200 micron. E. CD31 immunostaining of microvessels (black arrowheads) in vp from WT and EAF2^−/−^ FVB/NJ mice at 20 mos of age. Original magnification 10X, inset 40X. Scale bars indicate 200 micron in 10X, 50 micron in 40X.
(TIF)

Figure S2 **Effect of castration on C57BL/6J EAF2-deficient mice at age 19 weeks.** A. CD31 immunostaining of microvessels (black arrowheads) in transverse sections of ventral prostate lobes from wild-type (WT) and EAF2^−/−^ mice on a C57BL/6J background 14 days post-castration (Cx) at 19 weeks of age. Original magnification 10X, inset 20X. Scale bars indicate 200 micron. B. Ki-67 immunostaining (black arrowheads) in transverse sections of prostate anterior lobes from WT and EAF2^−/−^ C57BL/6J mice 14 days post-Cx at 19 weeks of age. Original magnification 20X. Scale bars indicate 200 micron. C. Caspase 3 immunostaining (black arrowheads) in transverse sections of prostate anterior lobes from WT and EAF2^−/−^ C57BL/6J mice 14 days post-Cx at 19 weeks of age. Original magnification 20X. Scale bars indicate 200 micron.
(TIF)

Figure S3 **EAF2 expression and CD34-positive microvessel density in matched normal adjacent prostate and prostate cancer tissue specimens.** A. Laser capture microdissection of prostate glandular epithelial cells for qPCR analyses. Scale bars indicate 50 micron. B. Immunostaining analysis of EAF2 and CD34-positive microvessels in prostate tissues. Original magnification 10X, inset 20X. Scale bars indicate 200 micron in 10X and 200 micron in 20X.
(TIF)
