# Supplementary figures and images for: Development of a Reactive Stroma Associated with Prostatic Intraepithelial Neoplasia in EAF2 Deficient Mice
Source: PLoS One. 2013 Nov 18;8(11):e79542. doi: 10.1371/journal.pone.0079542 (PMC3832612; doi:10.1371/journal.pone.0079542)

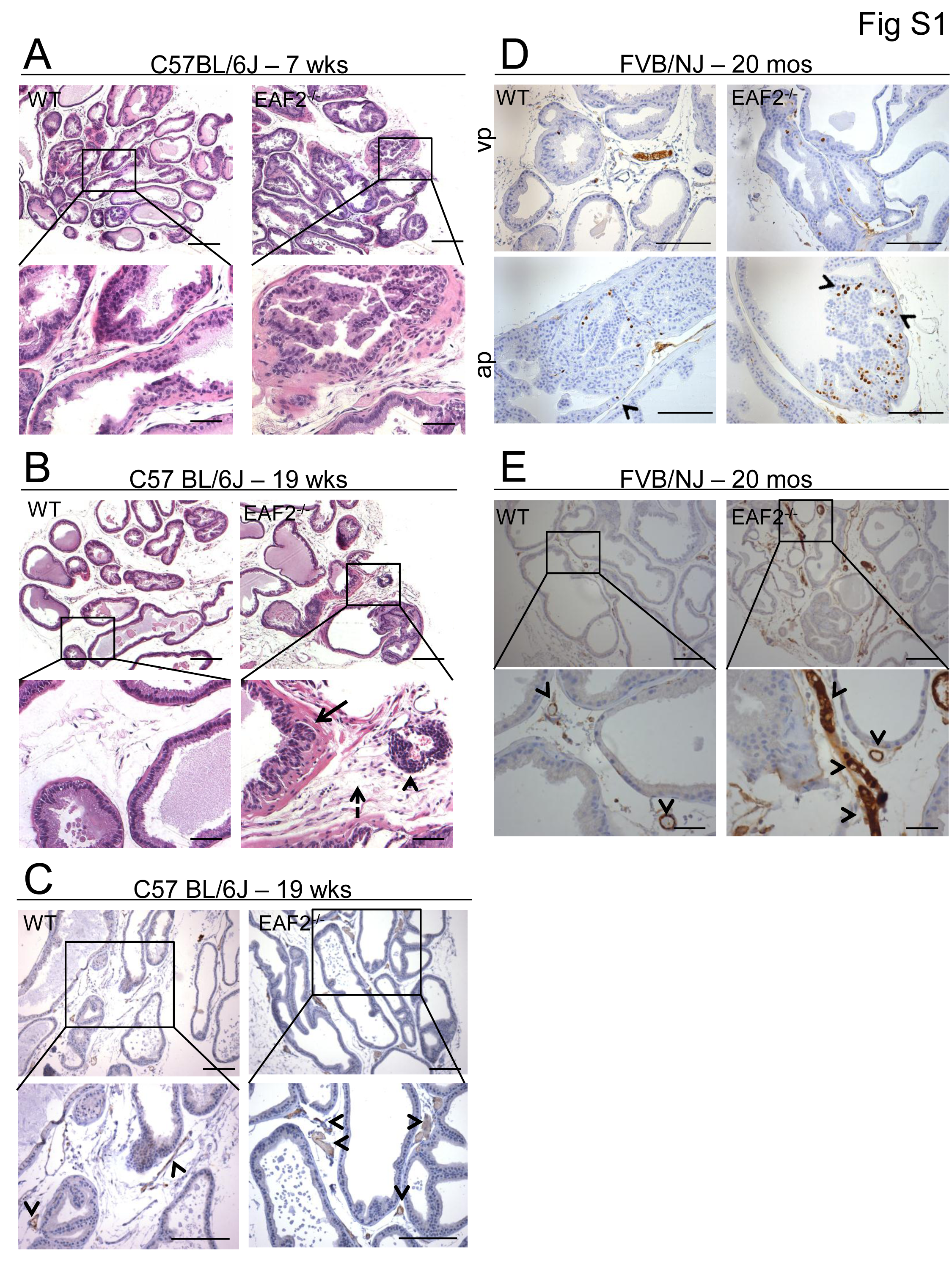

Supplement: Figure S1 — Effects of EAF2 loss on C57BL/6J and FVB/NJ mouse prostate histology. A. EAF2-deficiency induced stromal inflammation occurred as early as 7 wks in C57BL/6J mice. B. EAF2−/− murine prostates displayed prostate epithelial hyperplasia (black arrow, inset), evenly distributed mild stromal inflammation characterized by increased edema, lymphocytic infiltration, plasma cells, neutrophils, macrophages, mast cells and fibroblasts (dashed arrow, inset) compared to wild-type (WT) at age 19 wks. Microvessels (black arrowhead, inset) in the prostates of EAF2−/− mice were frequently associated with inflammation (black arrowhead, inset). Original magnification for A and B: 10X, inset 40X. Scale bars indicate 200 micron in 10X, 50 micron in 40X. C. CD31 immunostaining of microvessels (black arrowheads) in transverse sections of dorsal-lateral prostate lobes from wild-type (WT) control and EAF2−/− mice on a C57BL/6J background at 19 weeks of age. Original magnification for C: 10X, inset 20X. Scale bars indicate 200 micron. D. Ki-67 immunostaining (black arrowheads) in transverse sections of prostate ventral (vp) and anterior (ap) lobes from WT and EAF2−/− FVB/NJ mice at 20 mos of age. Original magnification 20X. Scale bar represents 200 micron. E. CD31 immunostaining of microvessels (black arrowheads) in vp from WT and EAF2−/− FVB/NJ mice at 20 mos of age. Original magnification 10X, inset 40X. Scale bars indicate 200 micron in 10X, 50 micron in 40X. (TIF) [file pone.0079542.s001.tif]

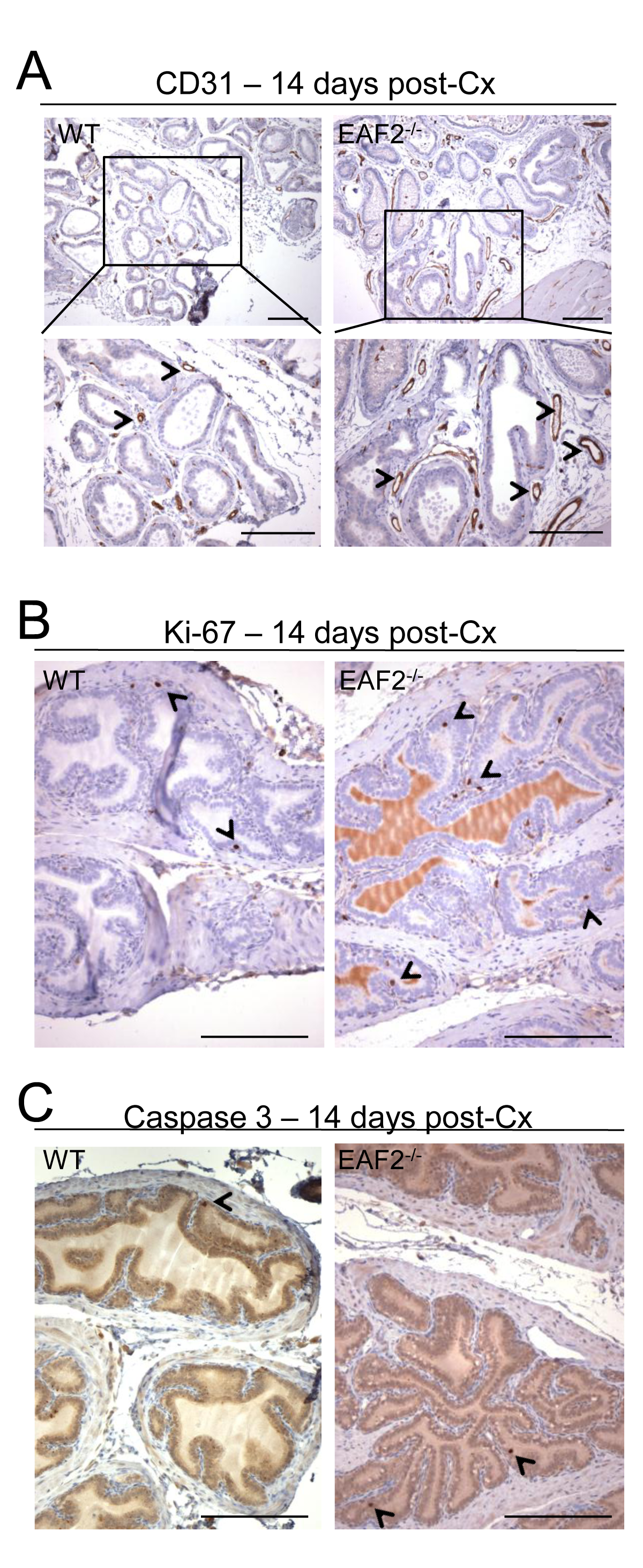

Supplement: Figure S2 — Effect of castration on C57BL/6J EAF2-deficient mice at age 19 weeks. A. CD31 immunostaining of microvessels (black arrowheads) in transverse sections of ventral prostate lobes from wild-type (WT) and EAF2−/− mice on a C57BL/6J background 14 days post-castration (Cx) at 19 weeks of age. Original magnification 10X, inset 20X. Scale bars indicate 200 micron. B. Ki-67 immunostaining (black arrowheads) in transverse sections of prostate anterior lobes from WT and EAF2−/− C57BL/6J mice 14 days post-Cx at 19 weeks of age. Original magnification 20X. Scale bars indicate 200 micron. C. Caspase 3 immunostaining (black arrowheads) in transverse sections of prostate anterior lobes from WT and EAF2−/− C57BL/6J mice 14 days post-Cx at 19 weeks of age. Original magnification 20X. Scale bars indicate 200 micron. (TIF) [file pone.0079542.s002.tif]

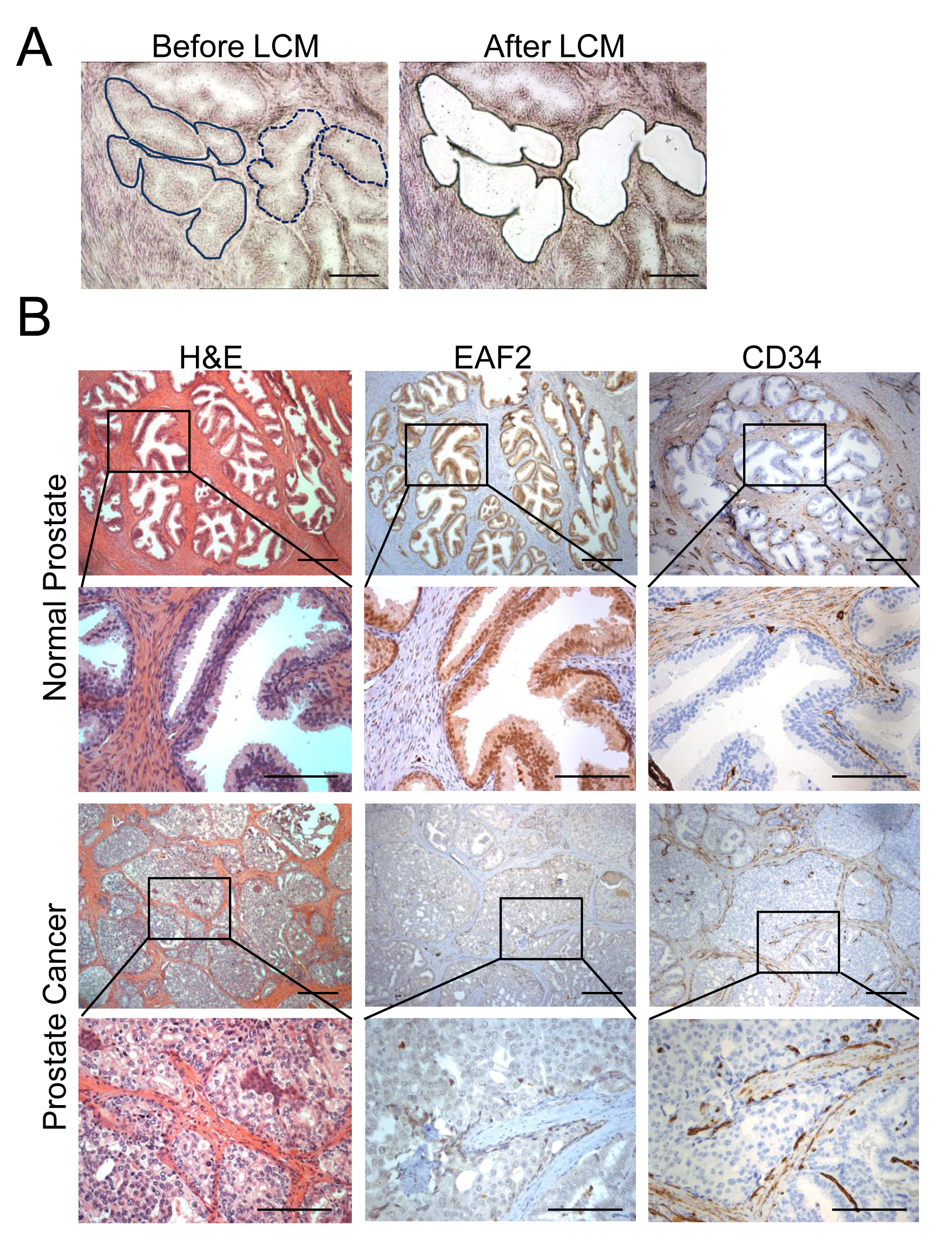

Supplement: Figure S3 — EAF2 expression and CD34-positive microvessel density in matched normal adjacent prostate and prostate cancer tissue specimens. A. Laser capture microdissection of prostate glandular epithelial cells for qPCR analyses. Scale bars indicate 50 micron. B. Immunostaining analysis of EAF2 and CD34-positive microvessels in prostate tissues. Original magnification 10X, inset 20X. Scale bars indicate 200 micron in 10X and 200 micron in 20X. (TIF) [file pone.0079542.s003.tif]
